# DNA repair inhibition by UVA photoactivated fluoroquinolones and vemurafenib

**DOI:** 10.1093/nar/gku1213

**Published:** 2014-11-20

**Authors:** Matthew Peacock, Reto Brem, Peter Macpherson, Peter Karran

**Affiliations:** Cancer Research UK London Research Institute, Clare Hall Laboratories, South Mimms, Herts. EN6 3LD, UK

## Abstract

Cutaneous photosensitization is a common side effect of drug treatment and can be associated with an increased skin cancer risk. The immunosuppressant azathioprine, the fluoroquinolone antibiotics and vemurafenib—a BRAF inhibitor used to treat metastatic melanoma—are all recognized clinical photosensitizers. We have compared the effects of UVA radiation on cultured human cells treated with 6-thioguanine (6-TG, a DNA-embedded azathioprine surrogate), the fluoroquinolones ciprofloxacin and ofloxacin and vemurafenib. Despite widely different structures and modes of action, each of these drugs potentiated UVA cytotoxicity. UVA photoactivation of 6-TG, ciprofloxacin and ofloxacin was associated with the generation of singlet oxygen that caused extensive protein oxidation. In particular, these treatments were associated with damage to DNA repair proteins that reduced the efficiency of nucleotide excision repair. Although vemurafenib was also highly phototoxic to cultured cells, its effects were less dependent on singlet oxygen. Highly toxic combinations of vemurafenib and UVA caused little protein carbonylation but were nevertheless inhibitory to nucleotide excision repair. Thus, for three different classes of drugs, photosensitization by at least two distinct mechanisms is associated with reduced protection against potentially mutagenic and carcinogenic DNA damage.

## INTRODUCTION

Cutaneous photosensitivity is a common side effect of drug treatments. It is a consequence of the ability of drugs or their metabolites to absorb energy from solar radiation (reviewed in (1)) and can be associated with a significantly increased risk of non-melanoma skin cancer (NMSC). Photosensitization can proceed *via* an excited chromophore (Type I photosensitization) and/or the formation of singlet oxygen (Type II) (2). Reactive oxygen species derived from photosensitization can damage nucleic acids and their precursors, proteins and membrane lipids.

**Figure 1.**
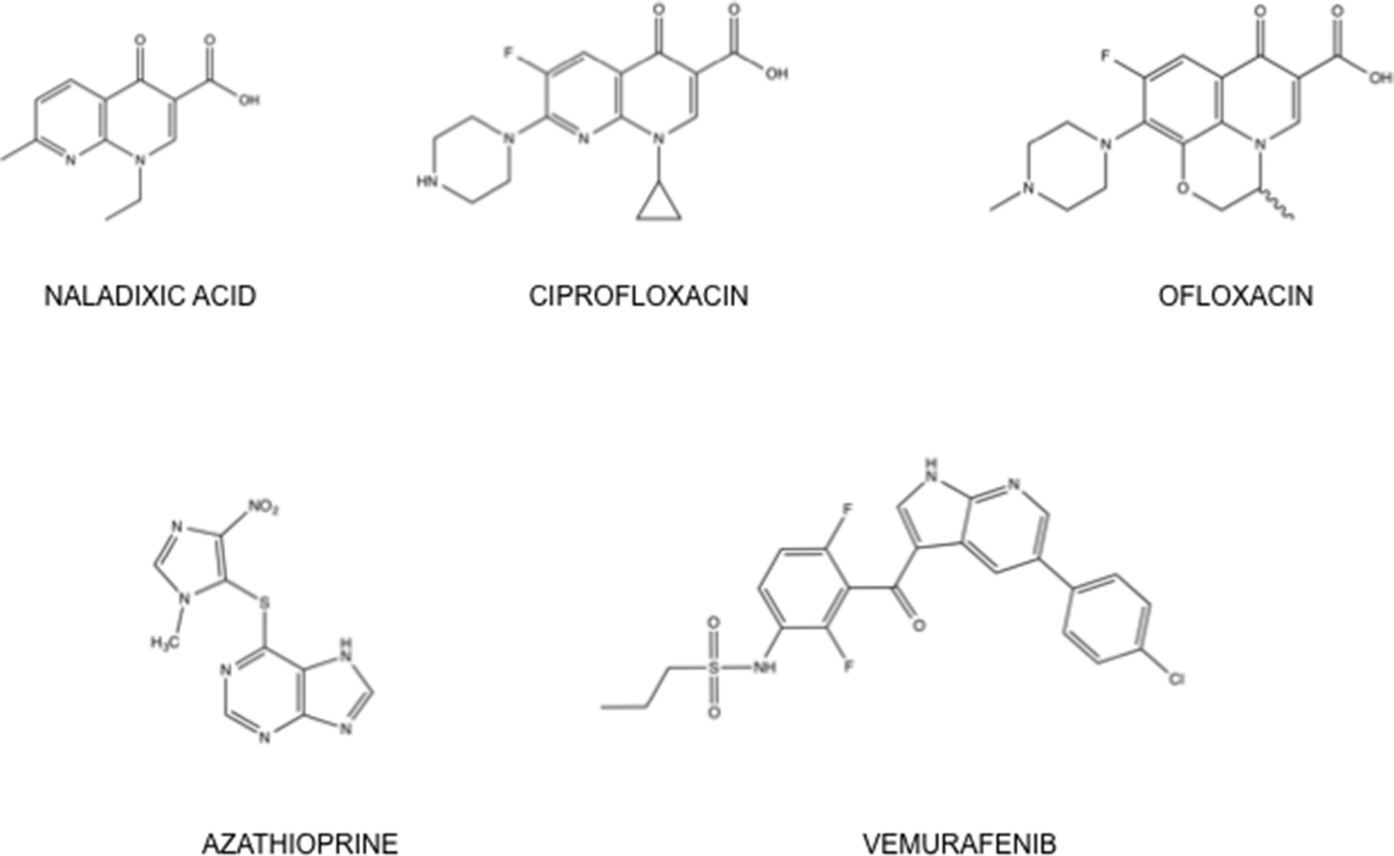
Structures of UVA photosensitizing drugs. Ciprofloxacin and ofloxacin are fluoroquinolones based structurally on naladixic acid. Azathioprine, an immunosuppressant and prodrug of 6-mercatopurine, is a source of DNA 6-TG. Vemurafenib is a BRAF inhibitor that selectively targets the Val600Glu mutant BRAF that is present in around 50% of melanomas.

The fluoroquinolone antibiotics are acknowledged photosensitizers (1) associated with a range of adverse cutaneous reactions (3,4). They are photocarcinogens in mice (5,6) and increase the risk of pre-malignant skin lesions in patients (7,8). Structurally based on naladixic acid (Figure 1) with a C6 fluorine substituent, fluoroquinolones inhibit DNA gyrase and topoisomerase IV that are essential for bacterial replication (9). They are also ultraviolet (UV) chromophores with absorbance peaks in both the UVB (280–315 nm) and UVA (315–400 nm) wavebands (10) and can interact with UV radiation to introduce DNA strand-breaks, DNA cyclobutane thymine dimers (T<>T CPDs) and oxidized bases by both Type I and Type II photosensitized reactions (11–13). Ciprofloxacin (Figure 1) is the most commonly prescribed fluoroquinolone in the United Kingdom followed by levofloxacin (the active enantiomer of ofloxacin) (www.hscic.gov.uk). Ciprofloxacin is more frequently associated with cutaneous side effects than ofloxacin (Figure 1) (14,15).

The antimelanoma drug vemurafenib (Figure 1), an inhibitor of the BRAF serine-threonine kinase involved in the control of cell proliferation, also causes cutaneous photosensitivity (16). Vemurafenib selectively targets the Val600Glu mutated BRAF that constitutively activates the MAP kinase (MAPK) signaling cascade in around 50% of metastatic melanomas (reviewed in (17)). Vemurafenib therapy is associated with a high incidence of squamous cell skin carcinoma (18,19). Its photosensitizing mechanism is unknown although it appears to be selective for UVA (16).

Azathioprine (Figure 1) is prescribed as an immunosuppressant in organ transplant patients and in the treatment of inflammatory bowel disorders. It is associated with an increased NMSC risk in both treatment contexts (20–22). Azathioprine causes selective UVA photosensitivity (23) and patients taking azathioprine accumulate the UVA chromophore 6-thioguanine (6-TG) in their DNA (24). In cultured human cells, DNA 6-TG acts as a Type I and Type II UVA photosensitizer. UVA activation of DNA 6-TG causes extensive DNA damage that includes oxidized forms of DNA 6-TG, 8-oxoG and DNA strand breaks (25). Type I reactions most likely underlie the formation of 6-TG/UVA-mediated DNA interstrand crosslinks and DNA-protein crosslinks (26–28). UVA activation of DNA 6-TG also causes widespread protein oxidation that diminishes DNA repair efficiency. In particular, the nucleotide excision repair (NER) pathway that removes the potentially mutagenic products of solar radiation, DNA 6–4 pyrimidine:pyrimidones (6–4 Py:Pys) and cyclobutane pyrimidine dimers (CPDs), is inhibited in cells treated with 6-TG and UVA (29). Since these photoproducts are implicated in the development of all forms of skin cancer (30–32), their possible impaired removal in a clinical context may have consequences for therapy-related skin cancer risk.

Thus, despite structural heterogeneity and differing therapeutic targets, all these drugs are photosensitizing skin carcinogens. Because the azathioprine surrogate DNA 6-TG is unusual in that it is incorporated into DNA, we compared its well-established effects with those of non-DNA-embedded photosensitizers. Here, we report that, like DNA 6-TG, the fluoroquinolones (ciprofloxacin and ofloxacin) and vemurafenib synergistically enhance UVA toxicity in cultured human cells. Although they are not incorporated into DNA, the effects of ciprofloxacin and ofloxacin resemble those of DNA-embedded 6-TG. They combine with UVA to cause extensive protein oxidation. The mechanism of vemurafenib photosensitization is different and involves less damage to DNA and to proteins. Despite these differences, each of these photosensitizers interacts with UVA to inhibit the removal of sunlight-associated, potentially mutagenic DNA lesions.

## MATERIALS AND METHODS

### Chemicals

6-TG, ciprofloxacin and ofloxacin were obtained from Sigma Aldrich. Vemurafenib was from Cayman Chemical and AlexaFluor 647® Hydroxylamine (FHA) from Life Technologies. PCNA (PC10) antibody was from Santa Cruz.

### Protein oxidation

Protein carbonylation was assayed as described previously (29,33). Briefly, extracts were incubated with 50 μg/ml FHA for 2 h at 37°C in phosphate buffered saline (PBS) and separated by polyacrylamide gel electrophoresis (PAGE). Proteins were visualized by Sypro Ruby staining and derivatized proteins by 633 nm fluorescence.

### Cell culture and survival

CCRF-CEM and HeLa cells were grown in RPMI or Dulbecco's modified Eagle's medium, respectively, supplemented with 10% FCS. For cytotoxicity measurements, CCRF-CEM cells that had been treated for 1 h or 24 h in growth medium were UVA irradiated in PBS. Following return to growth medium, the outgrowth of survivors was determined by trypan blue exclusion.

### UV irradiation

Cells were irradiated in PBSA. UVA irradiation was with a UVH 250W iron bulb (UV Light Technology Limited) at a dose rate of 0.1 kJ/m^2^/s. Maximum emission was 365 nm. UVC was delivered by a 254 nm UV light bulb in a Stratalinker UV Crosslinker (Stratagene) at a dose rate of ∼10 J/m^2^/s.

### DNA photoproduct determination

6–4 Py:Py and CPD photoproducts in DNA from treated cells were determined by enzyme-linked immunosorbent assay (ELISA) according to the suppliers' (Cosmo Bio Co) instructions.

### NER assays

NER was assayed in extracts prepared from treated cells as previously described (29,34)

## RESULTS

### UVA photosensitization

**Figure 2.**
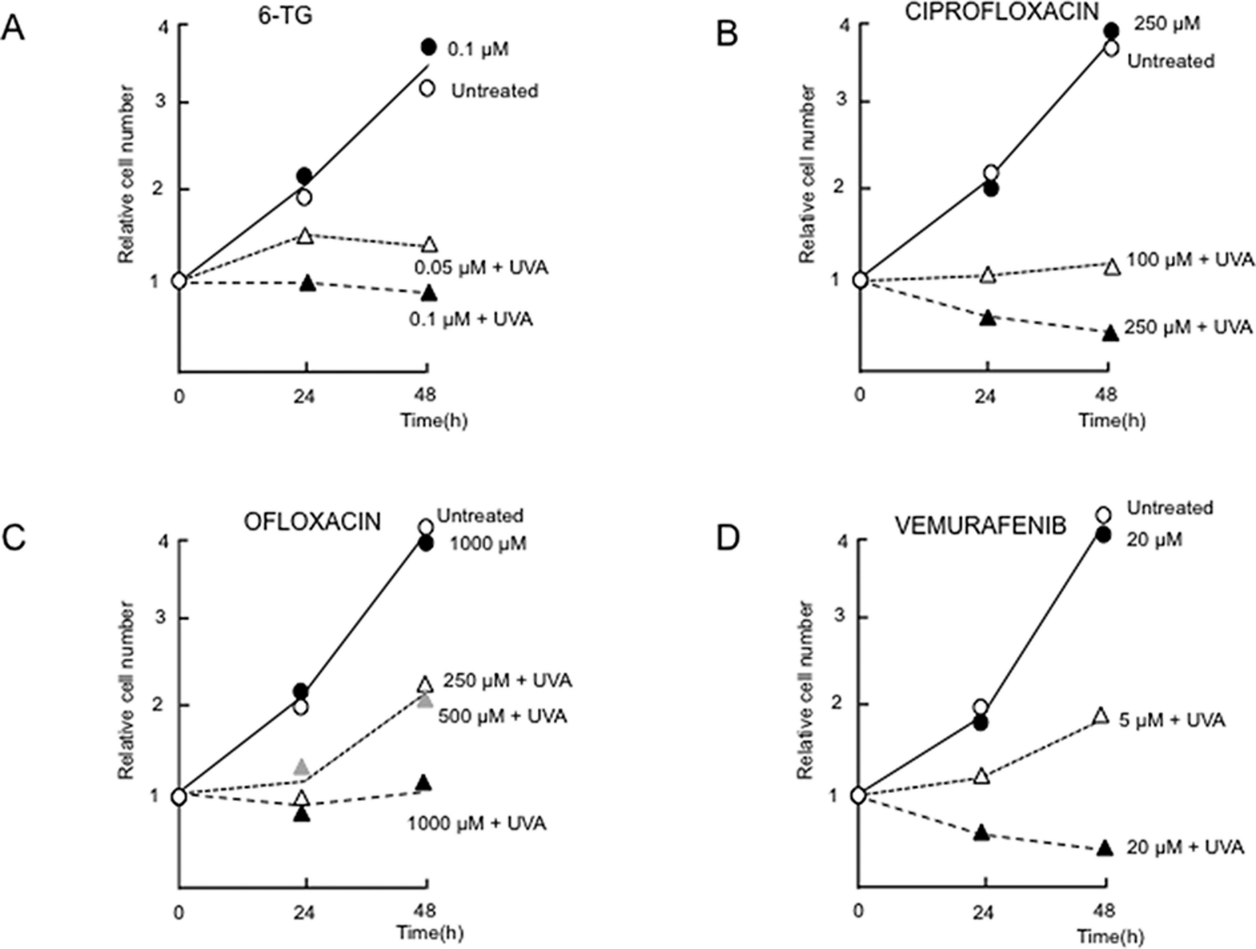
UVA-sensitized cytotoxicity. Exponentially growing CCRF-CEM cells that had been treated with 6-TG (0.05 or 0.1 μM for 24 h), ciprofloxacin (100 or 250 μM for 1 h), ofloxacin (250, 500 or 1000 μM for 1 h) or vemurafenib (5 or 20 μM for 24 h) were UVA irradiated (20 kJ/m^2^) in PBSA as indicated. Cells were returned to full growth medium without drug and live cells counted at the times shown. Cell counts are expressed relative to the starting cell number. Untreated cells received neither drug nor UVA. Untreated ∘; Drug alone at highest concentration •.

DNA 6-TG, ciprofloxacin, ofloxacin and vemurafenib all sensitized human CCRF-CEM cells to killing by UVA. Photosensitization was determined by monitoring cell outgrowth following UVA irradiation of drug-treated cells. 6-TG was introduced into DNA by growing cells for 24 h in medium containing non-toxic 6-TG concentrations. In agreement with previous observations in other human cell lines (24,35), DNA 6-TG and UVA were synergistically cytotoxic in CCRF-CEM cells (Figure 2A).

Ciprofloxacin and ofloxacin phototoxicity was determined after cells had been allowed to take up the drug for 1 h before irradiation. A dose of 20 kJ/m^2^ UVA caused complete inhibition of proliferation at ≥100 μM ciprofloxacin (Figure 2B) or >500 μM ofloxacin (Figure 2C). A 24-h treatment with ≥5 μM vemurafenib and 20 kJ/m^2^ UVA was also highly toxic (Figure 2D). Exposure to vemurafenib for 1 h also sensitized cells to UVA (Supplementary Figure S1), indicating that photosensitivity is most likely due to vemurafenib rather than its metabolites or its late effects on transcription. Toxicity was UVA-dependent in all cases and cell growth was unaffected by treatment with ciprofloxacin or ofloxacin (1 h, 1000 μM) or vemurafenib (1 or 24 h, 20 μM). In the absence of drug treatment, 20 kJ/m^2^ UVA was not toxic.

These findings confirm that the clinical photosensitizers DNA 6-TG, ciprofloxacin, ofloxacin and vemurafenib are all synergistically toxic with UVA in cultured human cells. Subsequent comparisons of their effects were performed at approximately equitoxic drug/UVA combinations.

### Protein oxidation

**Figure 3.**
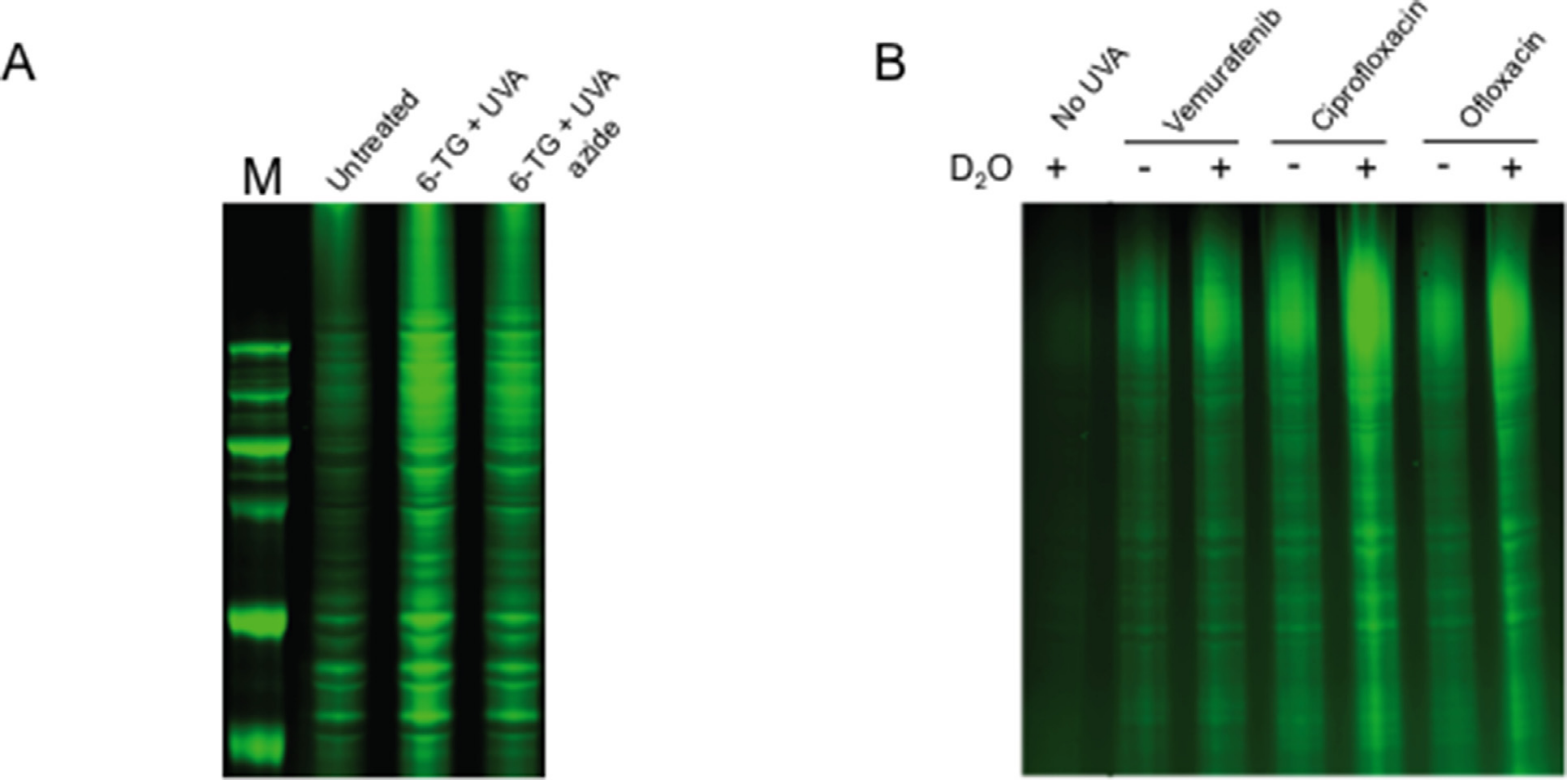
Photosensitized protein carbonylation. (A) CCRF-CEM cells were treated with 6-TG (0.6 μM, 24 h) and UVA irradiated (20 kJ/m^2^) in the presence or absence of azide (1 mM). Extracts were derivatized with AlexaFluor 647 hydroxylamine and separated by PAGE. Protein carbonyls were detected by fluorescence at 633 nm. M, molecular weight markers. (B) PAGE analysis of protein carbonyls in CCRF-CEM cells that had been treated with ciprofloxacin, ofloxacin (500 μM, 1 h) or vemurafenib (20 μM, 24 h) and UVA irradiated (20 kJ/m^2^) in PBS made in H_2_O or D_2_O as indicated.

Protein oxidation was examined by monitoring the formation of carbonyls. Proteins in extracts prepared from treated cells were derivatized with a carbonyl-reactive fluorophore (44), separated by PAGE and visualized by 633 nm fluorescence. UVA irradiation of cells containing DNA 6-TG induces extensive protein carbonylation (29). This is illustrated in Figure 3A which also shows that 6-TG/UVA-induced carbonylation is suppressed in the presence of azide, confirming that ^1^O_2_, a significant product of this interaction (36), is an important contributor to DNA 6-TG-mediated protein oxidation.

Ciprofloxacin and ofloxacin also enhanced protein carbonylation by UVA (Figure 3B). Protein oxidation was significantly enhanced when ciprofloxacin- or ofloxacin-treated cells were irradiated in D_2_O to enhance ^1^O_2_ reactivity (Figure 3B). UVA-dependent protein oxidation appears to be a characteristic of the fluoroquinolones and both norfloxacin and lomefloxacin increased carbonylation in a UVA-dependent fashion (Supplementary Figure S2A and B). Protein carbonylation by vemurafenib/UVA treatment was less extensive (Figure 3B) and was not detectably affected by D_2_O. In all cases, protein oxidation was UVA-dependent and ciprofloxacin, ofloxacin (500 μM) or vemurafenib (20 μM) did not increase carbonylation in unirradiated cells.

We conclude that ^1^O_2_-mediated protein oxidation is a feature of UVA photoactivated DNA 6-TG and the fluoroquinolones but is less extensive following vemurafenib photoactivation.

### PCNA modification

**Figure 4.**
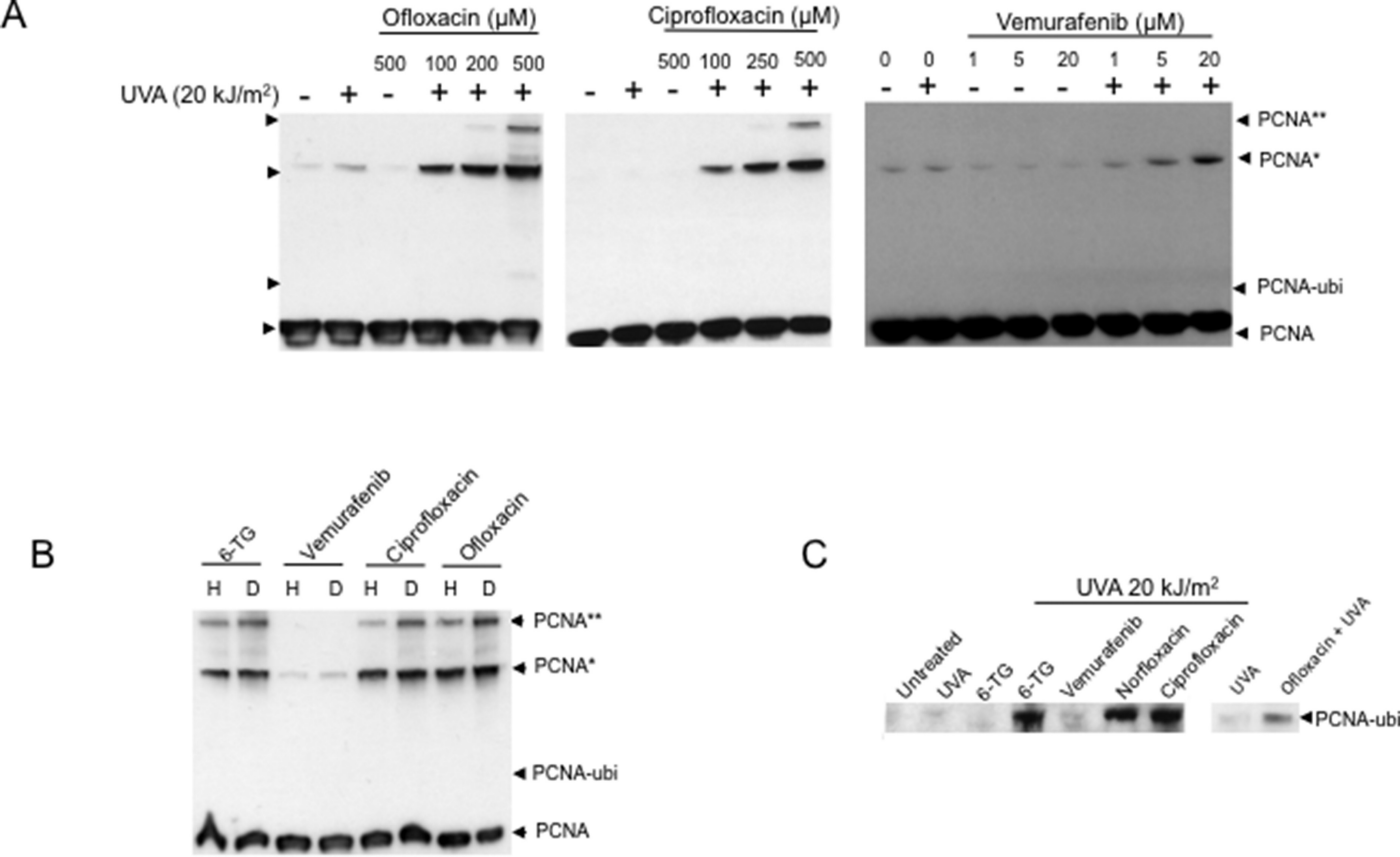
PCNA modification. (A) Extracts of CCRF-CEM cells treated as indicated (fluoroquinolones 1 h; vemurafenib 24 h) were analysed by western blotting and probed for PCNA. The positions of migration of the various PCNA forms are indicated. (B) ^1^O_2_-dependence of PCNA crosslinking. CCRF-CEM cells treated with 6-TG (0.6 μM, 24 h), ciprofloxacin, ofloxacin (500 μM, 1 h) or vemurafenib (20 μM, 24 h) were irradiated with UVA (20 kJ/m^2^) in PBSA made in H_2_O (H) or D_2_O (D) as indicated. PCNA was analysed by western blotting. (C) PCNA-ubi in western blots. Cells were treated with 6-TG (0.6 μM, 24 h), fluoroquinolones (500 μM, 1 h) or vemurafenib (20 μM, 24 h) and UVA irradiated. Western blots were exposed for longer to reveal PCNA-ubi.

Crosslinking of the 30 kDa subunits of the PCNA DNA replication and repair homotrimer is a sensitive indicator of ^1^O_2_-induced oxidative protein damage (37). DNA 6-TG significantly potentiates PCNA crosslinking by UVA (38) and induces two high molecular weight PCNA complexes. The first (PCNA*) migrates with an apparent mass of around 90 kDa. A more extensively crosslinked complex (PCNA**) is approximately twice this size. Ciprofloxacin and ofloxacin also sensitized UVA-mediated PCNA crosslinking and generated both PCNA* and PCNA** (Figure 4A). Consistent with the involvement of^1^O_2_, crosslinking was enhanced by irradiation in D_2_O (Figure 4B). PCNA crosslinking by vemurafenib/UVA was less efficient. Highly toxic combinations generated significantly less PCNA* and PCNA** was not detected. Vemurafenib/UVA-induced PCNA crosslinking was not detectably enhanced by irradiation in D_2_O (Figure 4B). PCNA complex formation was UVA-dependent and was not observed following drug treatment alone.

PCNA also undergoes monoubiquitination in response to the presence of replication-arresting DNA lesions (39). This modification (PCNA-ubi) is potentiated by 6-TG/UVA (38) (Figure 4C). UVA irradiation of ciprofloxacin- or ofloxacin-treated CCRF-CEM cells also induced PCNA-ubi (Figure 4C). Norfloxacin/UVA was included as a control in these experiments as a recognized source of replication-arresting DNA T<>T CPDs (11,13). In contrast, this PCNA modification was not detectable following vemurafenib/UVA treatment. The levels of PCNA-ubi were consistently lower in cells treated with ofloxacin/UVA compared to ciprofloxacin/UVA or DNA 6-TG/UVA. These observations indicate that UVA-activated ciprofloxacin, and at a lower efficiency ofloxacin, induce replication-blocking DNA damage. Vemurafenib is less effective in this regard.

**Figure 5.**
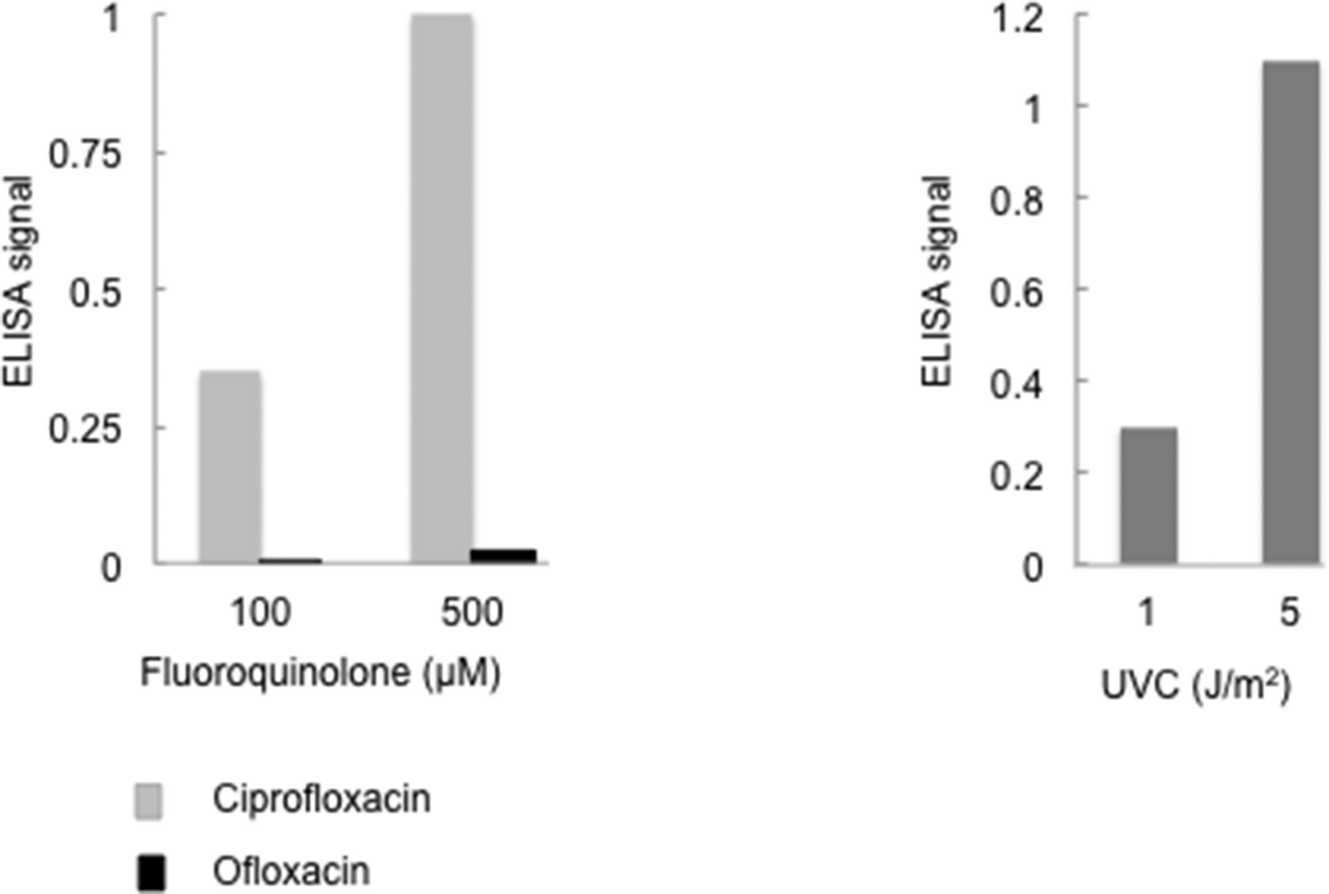
UVA-mediated CPD formation. DNA was extracted from CCRF-CEM cells that had been treated with ciprofloxacin or ofloxacin for 1 h at the indicated concentrations and UVA irradiated (20 kJ/m^2^) (left panel) or UVC irradiated (right panel). CPDs were analysed by ELISA. Ciprofloxacin + UVA 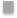
Ofloxacin + UVA ▪ UVC 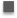.

Since norfloxacin photoactivation induces replication-arresting T<>T CPDs (11,12), we examined CPD formation in irradiated ciprofloxacin, ofloxacin, vemurafenib or 6-TG treated CCRF-CEM cells by ELISA. Figure 5 shows that ciprofloxacin/UVA treatment induces CPDs and that a combination of 500 μM ciprofloxacin and 20 kJ/m^2^ UVA is approximately equivalent to 5 J/m^2^ UVC in this regard. In agreement with its weaker induction of PCNA-ubi, ofloxacin/UVA induces many fewer CPDs. Following 500 μM ofloxacin and 20 kJ/m^2^ UVA, CPD levels were 1–2% of those induced by ciprofloxacin/UVA. No CPDs were detected in DNA from unirradiated or irradiated cells treated with 6-TG (0.05–0.25 μM, 24 h) or vemurafenib (20 μM, 24 h) and none of the drug/UVA combinations induced detectable 6–4 Py:Pys (data not shown). Thus, ciprofloxacin—and to a lesser extent ofloxacin—belong to the CDP-inducing group of fluoroquinolones which includes norfloxacin, lomefloxacin and enoxacin (11,12 and Supplementary Figure S2C).

### Inhibition of NER: *in vivo*

**Figure 6.**
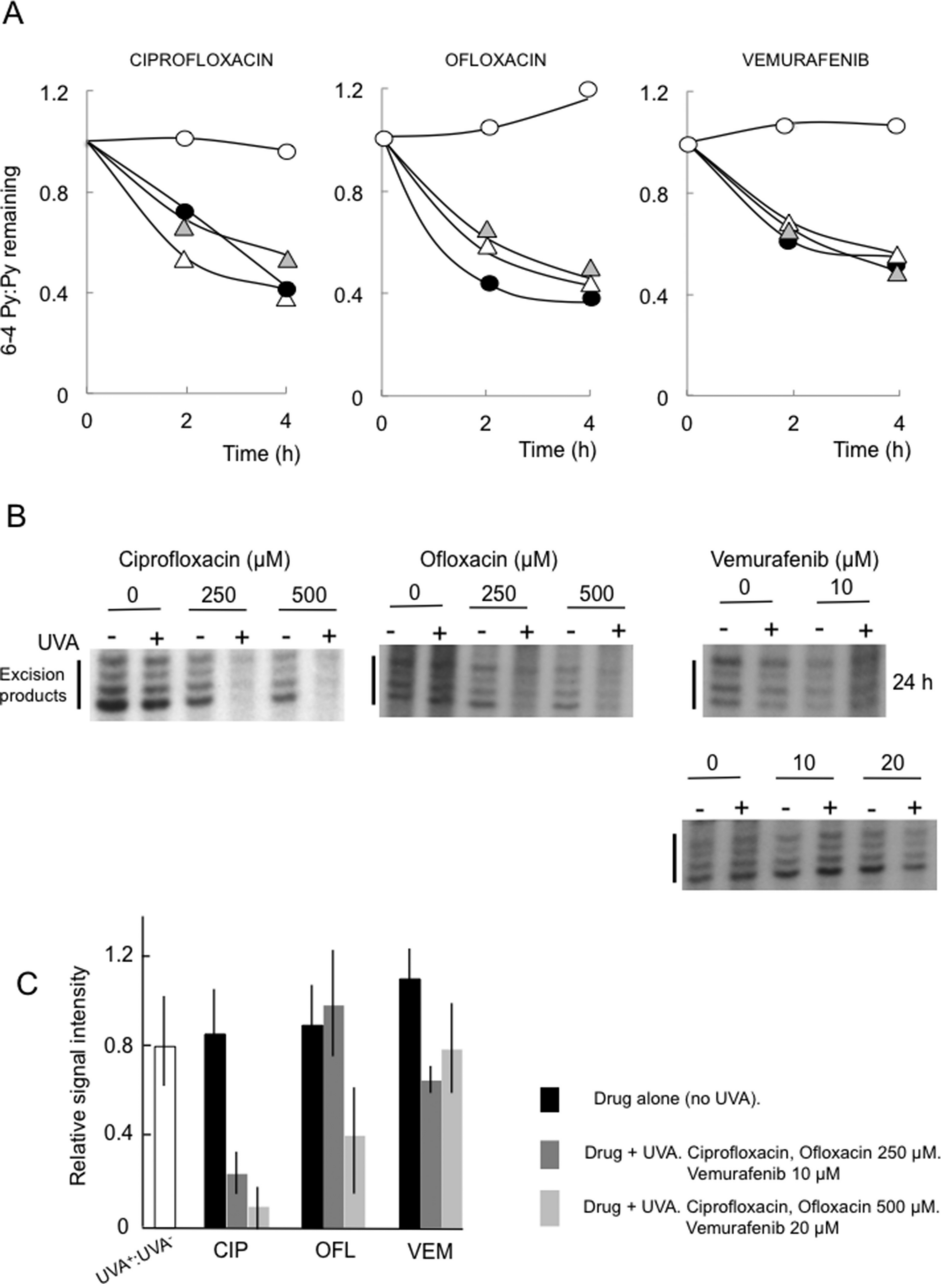
NER inhibition. (A) *In vivo* NER. CCRF-CEM cells treated with ciprofloxacin (500 μM, 1 h), ofloxacin (500 μM, 1 h) or vemurafenib (20 μM, 24 h) were irradiated with UVC (10 J/m^2^) followed by UVA (20 kJ/m^2^) as indicated. DNA was extracted at the times indicated following return to growth medium. 6–4 Py:Pys were analysed by ELISA. Untreated 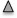
UVA alone Δ Drug alone • Drug + UVA ∘. (B) *In vitro* NER. Representative assays. Nuclear extracts were prepared from CCRF-CEM cells following 1 h (or 24 h as indicated) treatment with ciprofloxacin, ofloxacin or vemurafenib and UVA irradiation (20 kJ/m^2^). Extracts were incubated with the NER substrate and excised oligonucleotides (Excision products) were radiolabelled, separated by PAGE and detected by autoradiography. (C) Quantitation. Excision products were quantified by summing band intensities by GelDoc (Biorad). Integrated values for products generated by extracts from untreated cells were set to unity for each set of assays. Ordinate represents integrated values (mean ± SD) for excision products relative to this control. As there were no evident dose responses for drug treatment alone, data for the effects of drug (without UVA) represent combined values for both concentrations. For UVA^+^:UVA^−^, *n* = 9. For all other values *n* ≥ 3.

Exposure of cells containing DNA 6-TG to UVA inhibits NER (29). We examined the removal of 6–4 Py:Pys, a canonical NER substrate, in CCRF-CEM cells treated with ciprofloxacin, ofloxacin or vemurafenib and UVA-irradiated. The treated cells were irradiated with UVC to induce 6–4 Py:Pys which were measured by ELISA. In untreated cells or cells treated with drug or UVA alone, around 50% of 6–4 Py:Pys were removed in 2–4 h (Figure 6A). Ciprofloxacin/UVA and ofloxacin/UVA dramatically inhibited 6–4 Py:Py removal in the 4 h post-irradiation (Figure 6A). Vemurafenib/UVA was equally inhibitory. Inhibition was independent of the duration of vemurafenib exposure and UVA irradiation also suppressed NER after 1 h vemurafenib treatment (Supplementary Figure S3).

### Inhibition of NER: *in vitro*

An *in vitro* NER assay indicated that protein damage contributes significantly to NER inhibition by UVA-activated fluoroquinolones. Figure 6B shows that the excision of a substrate cisplatin intrastrand crosslink from circular DNA was impaired in extracts prepared from ciprofloxacin/UVA- and ofloxacin/UVA-treated CCRF-CEM cells. Surprisingly, NER was not significantly impaired in extracts prepared from UVA-irradiated cells treated with vemurafenib for 1 or 24 h (Figure 6B). Assays with extracts from similarly treated HeLa cells confirmed that ciprofloxacin/UVA and ofloxacin/UVA but not vemurafenib/UVA were inhibitory to NER (Supplementary Figure S4). These data indicate that, like DNA 6-TG/UVA (29), ciprofloxacin/ or ofloxacin/UVA treatment reduces NER capability by damaging DNA repair proteins. NER protein damage is not a major contributor to the inhibitory effects of vemurafenib/UVA on NER.

## DISCUSSION

We previously reported that the azathioprine surrogate DNA 6-TG is phototoxic in cultured human cells (24). The findings presented here demonstrate that the non-DNA-incorporated clinical photosensitizers, ciprofloxacin, ofloxacin and vemurafenib also significantly potentiate UVA toxicity. Like UVA-activated DNA 6-TG, the effects of fluoroquinolones are dependent on ^1^O_2_ and accompanying protein oxidation. Vemurafenib/UVA toxicity involves a different mechanism. Notwithstanding these mechanistic differences, each of the drugs inhibited NER in UVA-irradiated cells and an important aspect of our findings is that widely prescribed drugs that do not become incorporated into DNA sensitize cells to UVA-induced DNA repair inhibition.

Our experiments indicate that vemurafenib it is not a significant Type II photosensitizer and is a poor source of replication-arresting DNA lesions. It has been suggested (40) that increased porphyrin synthesis underlies vemurafenib photosensitization in a PDT-like reaction. In our experiments, short (1 h) vemurafenib treatment was sufficient to cause profound photosensitization. This observation (and similar findings in mouse cells (41)) appears to rule out porphyrin accumulation as a significant contributor to vemurafenib/UVA toxicity. We note, however, that vemurafenib itself is also extremely hydrophobic and, like porphyrins, liable to be sequestered in membranes. It is possible that vemurafenib phototoxicity, in common with that of photoactivated porphyrins, reflects its intracellular localization and damage to membranes rather than to other cellular components.

The fluoroquinolones and 6-TG are partial Type II UVA sensitizers that induce typical oxidative DNA damage including DNA 8-oxoG and strand breaks (10,25,42). Neither lesion is an efficient inhibitor of replication, however, and would not provoke PCNA ubiquitination. Our findings place ciprofloxacin in the subgroup of fluoroquinolones that photosensitize T<>T (11) but not 6–4 Py:Py formation. Ofloxacin/UVA is confirmed as being much less efficient in this regard (11). The different effects of UVA-activated ciprofloxacin and ofloxacin on PCNA monoubiquitination are therefore consistent with the induction of replication-blocking CPDs.

UVA activation of DNA 6-TG and the fluoroquinolones caused extensive protein carbonylation that was at least partly ^1^O_2_-dependent. It also induced^1^O_2_-dependent PCNA crosslinking. The functional importance of protein damage was revealed by the reduced NER activity in extracts from fluoroquinolone/UVA-treated cells. In this regard, the effects of photoactivated ciprofloxacin and ofloxacin again replicate those of DNA 6-TG/UVA which has been shown to damage RPA, an essential NER factor (29). Consistent with NER inhibition *in vitro*, UVA irradiation inhibited the excision of canonical UV-induced DNA photoproducts in ciprofloxacin- and ofloxacin-treated cells. Based on the similar effects of photoactivation of these two fluoroquinolones and DNA 6-TG, we suggest that protein damage is a significant contributor to NER inhibition by UVA-activated ciprofloxacin and ofloxacin.

Extracts from vemurafenib/UVA-treated cells retained apparently unaltered NER capability, an observation that is consistent with the induction of relatively low levels of protein oxidation. Vemurafenib nevertheless efficiently sensitized UVA-dependent inhibition of NER in intact cells. It seems likely therefore that the effects of vemurafenib/UVA on NER reflect cellular damage other than protein oxidation. We cannot at present exclude the possibility that these findings reflect the inherent artificiality of the *in vitro* assay. The maintenance of high protein concentrations and the presence of reducing agents during extract preparation may artificially normalize suboptimal levels of active NER proteins. In addition, the simple plasmid substrate used in the *in vitro* assay does not reflect the complexities of lesion recognition in chromatin or events downstream of lesion excision during NER in intact cells.

Notwithstanding their different mechanisms of NER inhibition, all the drugs we examined combined with UVA to inhibit the removal of canonical UVB photoproducts in intact cells. This observation has implications for skin cancer risk. NMSC is associated with photosensitizing therapies. The high NMSC incidence in azathioprine-immunosuppressed organ transplant patients or inflammatory bowel disorder sufferers is well-established (20–22). Ciprofloxacin and ofloxacin are both photocarcinogens in mice (5,6) and fluoroquinolones may be linked to an increased skin cancer risk (7,8). Verumafenib is associated with the rapid development of NMSCs (18). The incidence of these therapy-related NMSCs is consistent with at least two separate mechanisms that are reflected in our findings. The first mechanism is common to azathioprine and the fluoroquinolones. The skin cancer incidence in azathioprine patients is maximal 5 to 10 years post-transplant (43). The persistence of potentially mutagenic DNA lesions induced by sunlight UVB is a known long-term risk factor for skin carcinogenesis and this extended timescale of NMSC development is consistent with the acquisition of transforming mutations. We suggest that the NMSC risk in patients taking azathioprine or fluoroquinolones is increased by the attenuated NER of potentially mutagenic sunlight-induced DNA damage. This effect is exaggerated in azathioprine patients because they receive lifelong treatment. Generally briefer, antibiotic treatments would be expected to entail a proportionally reduced NMSC risk. Prophylactic ciprofloxacin, particularly in the context of immunosuppressed organ transplant patients (44) might, however, significantly increase this risk.

Vemurafenib-associated squamous cell carcinoma (18,19) clearly arises by a different mechanism. NMSC development is characteristically rapid and tumors can appear within weeks of starting vemurafenib therapy—a time scale that is clearly incompatible with the acquisition of mutations. Vemurafenib-associated NMSCs frequently contain mutated *RAS* (45). It seems that vemurafenib treatment permits the clonal expansion of sun-damaged skin cells with a preexisting *RAS* mutation. Selection may occur at the level of intracellular signaling cascades and be independent of the interaction of vemurafenib with UVA. Vemurafenib/UVA-induced inhibition of NER will, however, have implications for NMSC development in the longer term.

In summary, reduced NER efficiency is a common feature of UVA photosensitization by azathioprine, the fluoroquinolones and vemurafenib. For azathioprine and the fluoroquinolones, attenuated NER reflects damage to DNA repair proteins. Vemurafenib photosensitization occurs by a different mechanism that involves less extensive DNA and protein damage. In the long term, a reduced efficiency of NER increases the likelihood of mutation and of developing NMSC. Photosensitivity is a common clinical side effect and other drugs may also increase NMSC risk by interfering with DNA repair.

## SUPPLEMENTARY DATA

Supplementary Data are available at NAR Online.

SUPPLEMENTARY DATA
