# Supplementary material for: DNA repair inhibition by UVA photoactivated fluoroquinolones and vemurafenib
Source: Nucleic Acids Res. 2014 Nov 20;42(22):13714–22. doi: 10.1093/nar/gku1213 (PMC4267641; doi:10.1093/nar/gku1213)
Supplement: SUPPLEMENTARY DATA [file supp_42_22_13714__index.html]

DNA repair inhibition by UVA photoactivated fluoroquinolones and vemurafenib — DNA repair inhibition by UVA photoactivated fluoroquinolones and vemurafenib — SUPPLEMENTARY DATA 

# DNA repair inhibition by UVA photoactivated fluoroquinolones and vemurafenib

## SUPPLEMENTARY DATA

**Files in this Data Supplement:**

- SUPPLEMENTARY DATA
