# Supplementary material for: DNA repair inhibition by UVA photoactivated fluoroquinolones and vemurafenib
Source: Nucleic Acids Res. 2014 Nov 20;42(22):13714–22. doi: 10.1093/nar/gku1213 (PMC4267641; doi:10.1093/nar/gku1213)
Supplement: SUPPLEMENTARY DATA [file supp_gku1213_nar-02566-f-2014-File008.pdf]

Supplementary Figure 1 Growth inhibition by vemurafenib (1 h treatment)/UVA

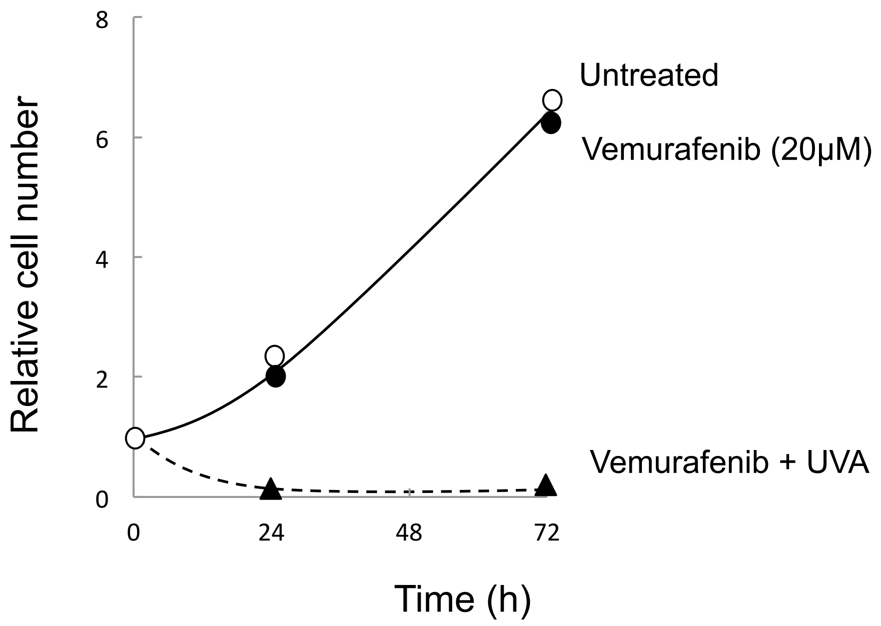


CCRF-CEM cells treated for 1 h with 20 μM vemurafenib were UVA-irradiated (20 kJ/m$^2$) in PBSA as indicated. Irradiated cells were returned to full growth medium without drug and live cells counted at the times shown. Cell counts are expressed relative to the starting cell number. Untreated cells received neither drug not UVA.

Supplementary Figure 2 Effects of Lomefloxacin & Norfloxacin

A

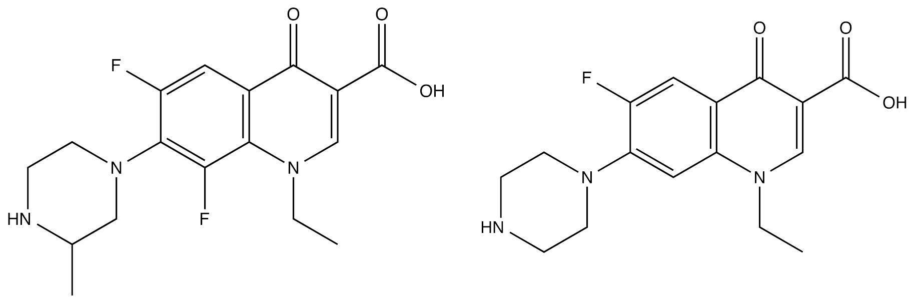


Lomefloxacin Norfloxacin

B

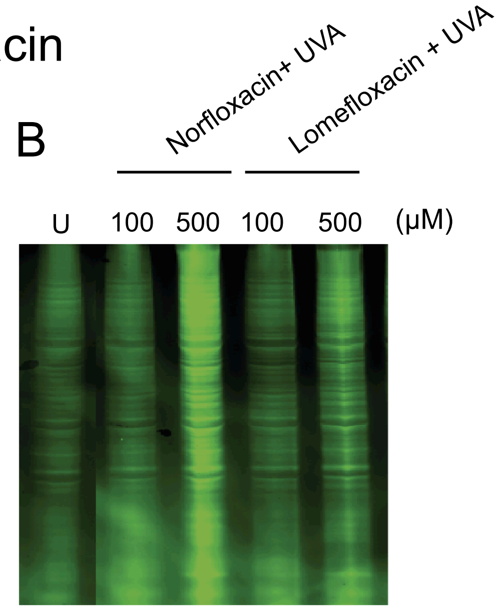


C

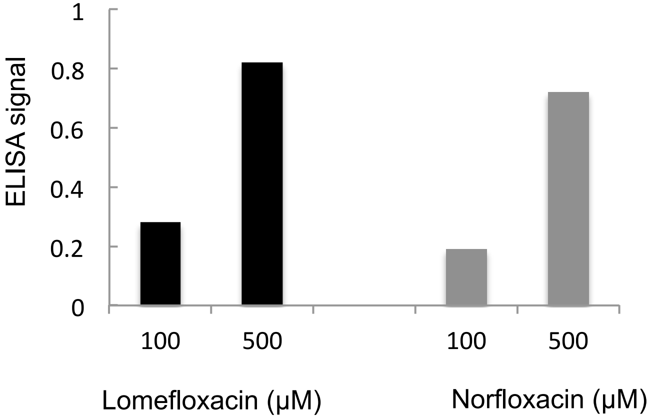


A Structures of lomefloxacin and norfloxacin

B Protein carbonylation Nuclear extracts were prepared from UVA irradiated (20 kJ/m$^2$) CCRF-CEM cells that had been treated (1 h) with norfloxacin or lomefloxacin as indicated. Protein carbonyls were derivatized with AlexaFluor 647 hydroxylamine (Poon, H. F. *et al. Biol. Proced. Online* **9**, 65-72 (2007)). Extracts (10 μg) were separated by PAGE and protein carbonyls were detected by fluorescence at 633 nm. U = untreated.

C UVA-dependent CPD induction. DNA was extracted from CCRF-CEM cells that had been treated (1 h) with the concentrations of lomefloxacin or norfloxacin indicated and UVA irradiated (20 kJ/m$^2$). CPDs were measured by ELISA.

Supplementary Figure 3 NER in UVA irradiated cells treated with vemurafenib for 1 h

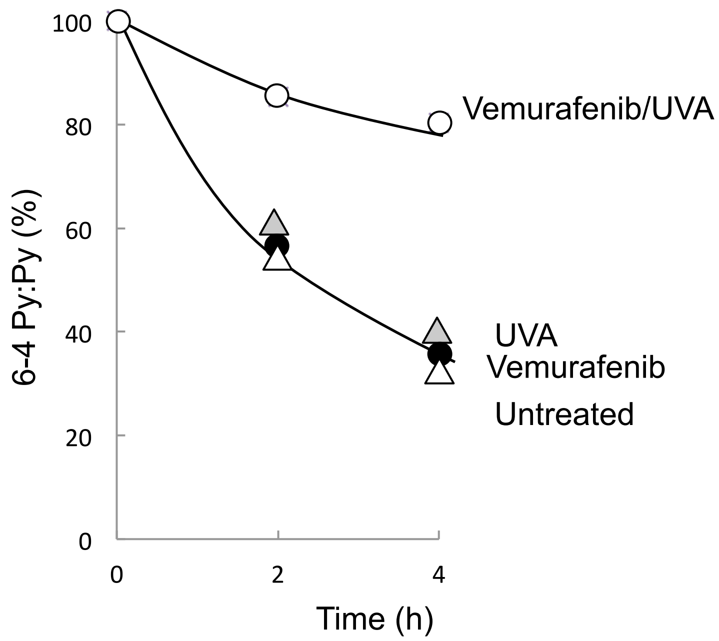


CCRF-CEM cells treated for 1 h with 20 μM vemurafenib were irradiated with UVA (20 kJ/m$^2$) and UVC (20 J/m$^2$). DNA was extracted at the times indicated and 6-4 Py:Py levels were measured by ELISA.

Supplementary Figure 4 NER *in vitro* - HeLa cells

A

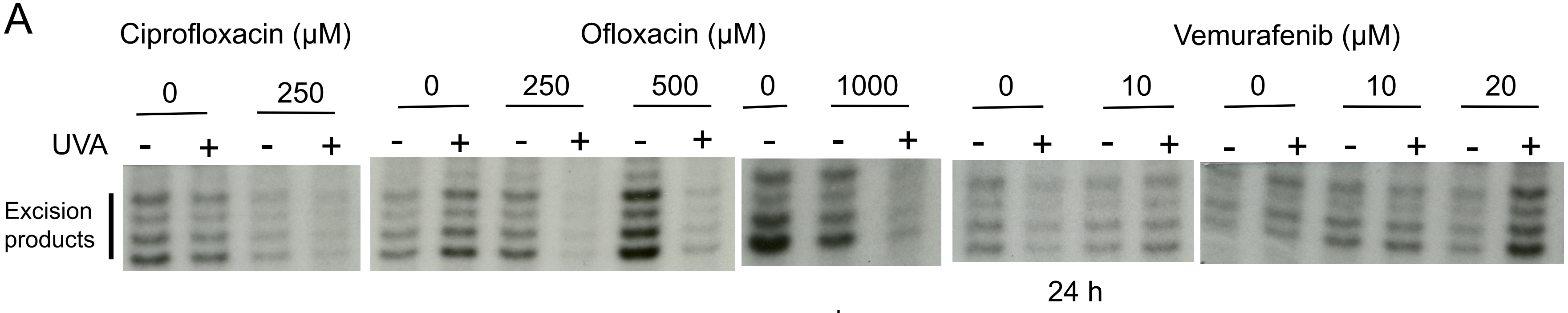


B

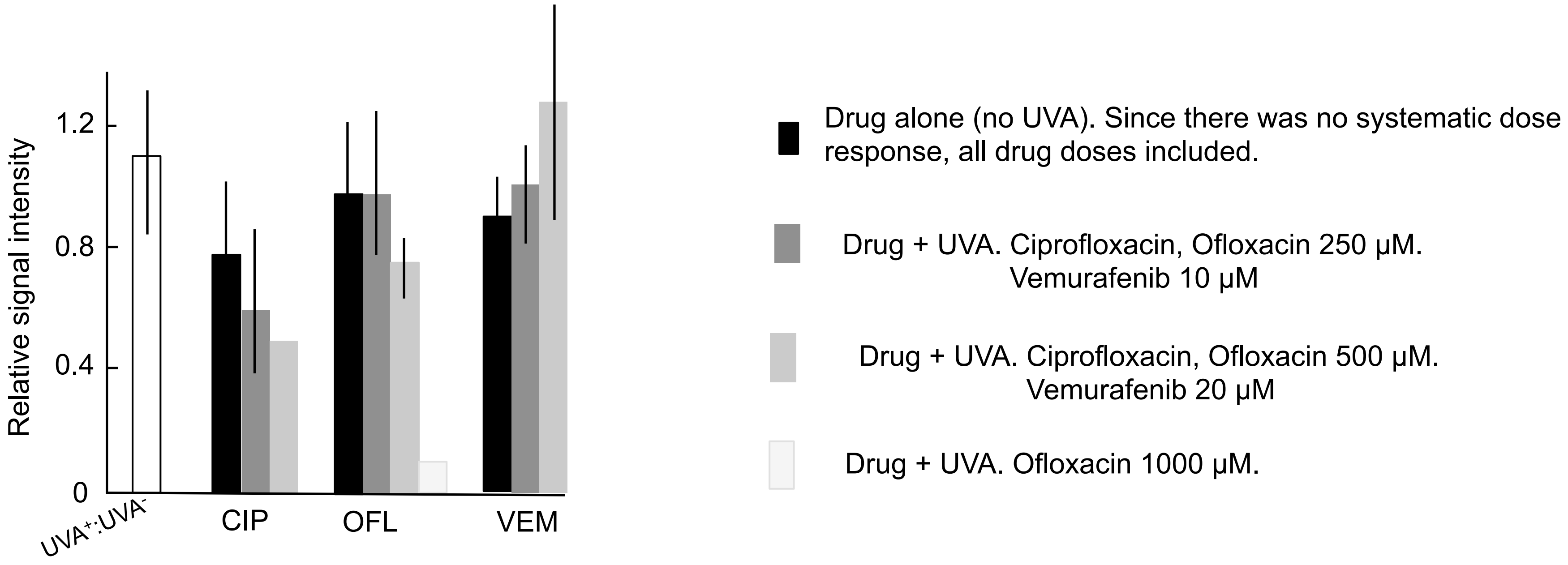


A Representative assays. HeLa cells treated for 1 h (or 24h as indicated) with drugs were irradiated with UVA (20 kJ/$m^2$) as shown. NER was assayed in nuclear extracts prepared immediately after irradiation. 27-31 nt excision products shown by bar.

B Excision products were quantified by summing band intensities by GelDoc (Biorad). Integrated values for products generated by extracts from untreated cells were set to unity for each set of assays. Ordinate represents integrated excision product band intensity values (Mean ± SD) relative to this control.

For $UVA^+$:$UVA^-$, n = 11. For other values n ≥ 3. Where no error bar is shown, value is mean of two determinations.
